# A Novel Eco-Friendly Process for the Synthesis and Purification of Ascorbyl-6-Oleates

**DOI:** 10.3390/foods14010070

**Published:** 2024-12-30

**Authors:** Ha-Eun Ji, Se-Young Kim, Heejin So, Vivian Prayitno, Ki-Teak Lee, Jung-Ah Shin

**Affiliations:** 1 Department of Food Science and Technology, Chungnam National University, 99 Daehak-ro, Yuseong-gu, Daejeon 34134, Republic of Korea; haean4395@cnu.ac.kr (H.-E.J.); seyoungkim@cnu.ac.kr (S.-Y.K.); vivianprayitnoo@gmail.com (V.P.); 2 Department of Human Nutrition, Food and Animal Sciences, University of Hawaii at Manoa, 1955 East-West Road, Honolulu, HI 96822, USA; heejinso@hawaii.edu; 3 Department of Marine Bio Food Science, Gangneung-Wonju National University, 7 Jukheon-gil, Gangneung 25457, Gangwon-do, Republic of Korea

**Keywords:** ascorbyl-6-O-esters, enzymatic esterification, enzymatic transesterification, recrystallisation, purification

## Abstract

Commercial ascorbyl-6-O-esters (AEs) are composed of saturated fatty acids with relatively high melting points, resulting in limited solubility in lipophilic media. Therefore, a lipase-catalysed synthesis and purification method for ascorbyl-6-O-oleate (AO) was proposed in this study. The esterification synthesis (i.e., bonding of oleoyl group to ascorbic acid) rate was 19.7% using acetone as the reaction solvent. The transesterification synthesis (i.e., exchange of acyl group with oleic acid (OA) in ascorbyl-6-O-palmitate (AP)) rate increased to 73.8% (AP:OA = 1:3, molar ratio). The esterification product was purified sequentially by liquid–liquid extraction using ethyl acetate and water, followed by hexane and acetonitrile, resulting in 94.8 area% AO confirmed by HPLC. When acetonitrile was replaced with 90% methanol, AO achieved 97.2 area%. Similarly, the transesterification product showed 94.3 area% AEs (AP:AO = 8.9:91.1) after recrystallisation and liquid–liquid extraction. Finally, all purified AO revealed peaks corresponding to the hydroxyl groups at the C-2 and C-3 carbons (11.10 and 8.41 ppm, ^1^H-NMR), whereas OA selectively esterified at the C-6 carbon (^13^C-NMR). FT-IR confirmed the presence of the ester bond (1733 cm^−1^) and olefin structure (3006 cm^−1^) of OA, and LC-ESI-MS/MS identified AO peaks at *m/z* 439.3. DSC analysis showed broad endothermic curves at 23.1–46.7 °C when the purified AO samples were pre-cooled at −25 °C.

## 1. Introduction

Antioxidants (i.e., reducing agents) are used to prevent the oxidation of compounds in foods and cosmetics. Synthetic antioxidants, such as butylated hydroxyanisole (BHA) and butylated hydroxytoluene (BHT), exhibit strong antioxidant activity and can be produced at a relatively low cost. However, they have been linked to health issues such as liver damage or possible oncogenicity [1,2]. Thus, there have been increasing efforts to explore natural antioxidants and their derivatives as alternatives to synthetic antioxidants [3,4].

L-ascorbic acid (AA), one of the best-known natural antioxidants, is used often in various industries. However, it is highly hydrophilic and readily oxidised when exposed to air or light, restricting its use to hydrophobic products [1,2,5]. To overcome these limitations, various AA derivatives have been studied [6,7,8]. Representative AA derivatives include ascorbyl-6-O-esters (AEs), which are synthesised by attaching an acyl group to AA to increase lipophilicity and impart antioxidative properties. Different AE derivatives can be synthesised depending on the type of acyl groups; however, in Korea, only ascorbyl-6-O-palmitate (AP) and ascorbyl-6-O-stearate are listed in the Food Additives Code. These compounds are known to be effective alternatives to synthetic antioxidants and exhibit antibacterial activity without posing health threats to humans [2].

AEs have not only antioxidant properties but also an amphiphilic structure, making them suitable for use as emulsifiers in various systems (bulk oil media, emulsions, liposomes, etc.) [9]. In emulsion systems, ensuring the oxidative stability of lipids within fat droplets is critical, as oxidation primarily occurs at the interface between oil and water. Therefore, when antioxidants such as AEs are positioned at this interface, they can effectively induce antioxidative effects on substances within the emulsion core [10,11,12]. Recently, when AP was used as an ionic surfactant, it also helped stabilise nanocarriers by increasing the negative zeta potential [13]. Because nanocarriers are a suitable system for the delivery of drugs and nutrients, AP could be an important component for drug delivery applications. Hence, AEs may be commercially useful beyond their typical application as lipophilic antioxidants.

When commercially manufactured, AEs are primarily synthesised utilising an acid catalyst such as sulfuric acid. However, oxidation, degradation, and rearrangement of AA results in a low synthesis yield, and tedious isolation is required to obtain a high-purity product [1,9,14]. In addition, unlike saturated fatty acids, unsaturated fatty acids do not easily react with AA in concentrated sulfuric acid due to their double bonds, leading to the formation of byproducts that are difficult to separate [15,16]. Conversely, enzymatic synthesis can be performed under mild conditions and reduces the formation of byproducts owing to the high positional selectivity of enzymes, thus overcoming the limitations of chemical synthesis [1,14,15,17].

For the enzymatic synthesis of AEs, the reaction conditions are a crucial consideration, including factors such as temperature, enzyme type and used amount, reaction solvent, and acyl donor type [15,16,17,18,19,20,21,22,23,24]. According to previous studies, the most suitable enzyme for synthesising AEs is Novozym^®^ 435, because it exhibits a higher initial reaction rate, yield, and productivity than other enzymes [18]. Various reaction solvents have been explored as well. Stojanović et al. reported the rates of synthesis with different reaction solvents and found that, under the same conditions, the yield was ∼20% in acetone and 50% in t-butanol [9], while Viklund et al. reported a maximum synthesis yield of 87% in t-amyl alcohol [15]. In contrast, despite reported low synthetic yields for AEs, acetone is a promising solvent for the development of an economical industrial enzymatic process. It is inexpensive and a safe, recommended solvent for industrial uses; additionally, acetone has a low boiling point and viscosity, making it easy to handle [9,25].

In this study, we focused on an AE derivative, namely ascorbyl-6-O-oleate (AO), which has not yet been commercialised. Commercially used AEs typically have saturated fatty acids in their acyl groups, resulting in low solubility for fat-soluble substances owing to their high melting point [9,14]. In contrast, AO contains unsaturated fatty acids with double bonds in the acyl groups, which can improve solubility and expand its applicability as an antioxidant and emulsifier in food, cosmetic, and pharmaceutical formulations.

Thus, we studied environmentally friendly enzymatic processes to increase the synthetic yield of AO using acetone as the reaction solvent. In addition, a purification method through liquid–liquid extraction and re-crystallisation was proposed to obtain high-purity AO from the reaction product.

## 2. Materials and Methods

### 2.1. Materials

AA, oleic acid (OA), and a molecular sieve (4 Å, 10–18 mesh) for AO synthesis were purchased from Sigma-Aldrich Chemical Co. (St. Louis, MO, USA). For OA, we used a technical-grade 90% composition of the three main fatty acids by gas chromatography-flame ionisation detection (GC-FID), as follows (area%): 89.50% OA, 5.43% linoleic acid, and 2.30% stearic acid. The solvents used in the synthesis and purification, including acetone, ethyl acetate (EA), water, hexane, and acetonitrile (ACN), were purchased from Daejung Co. (Incheon, Korea), and Novozym^®^ 435 enzyme (10,000 PLU/g) used in the reaction was purchased from Novozymes (Bagsvaerd, Denmark). AA 6-palmitate was purchased from Sigma-Aldrich Chemical Co. Triundecanoin used as an internal standard for the gas chromatography (GC) was purchased from Nu-chek Prep, Inc. (Elysian, MN, USA). Thin layer chromatography (TLC) silica gel F_254_ was purchased from Merck Co. (Darmstadt, Germany), and anhydrous sodium sulfate was purchased from Junsei Chemical Co., Ltd. (Tokyo, Japan). Dimethyl sulfoxide-d6 (DMSO-d6), used for nuclear magnetic resonance (NMR) spectroscopy, was purchased from Sigma-Aldrich Chemical Co. (St. Louis, MO, USA).

### 2.2. Synthesis and Purification of AO by Lipase-Catalysed Esterification

**Figure 1:**
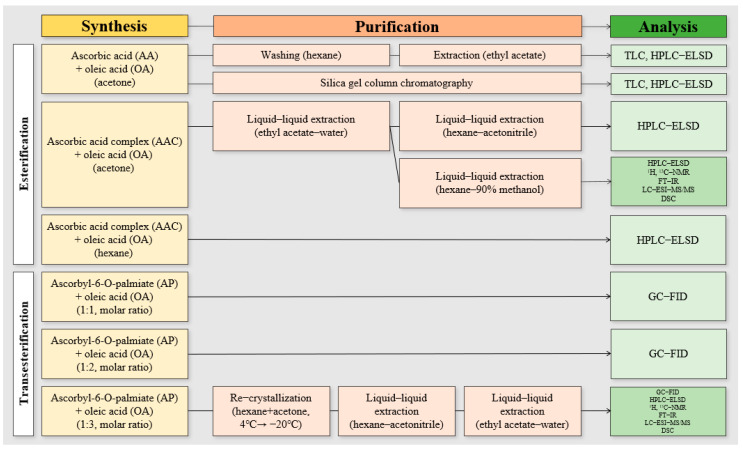
Scheme of the experimental design.

The scheme of the synthesis, purification, and analysis processes conducted in this study is shown in Figure 1.

#### 2.2.1. Synthesis of AO Using AA

**Figure 2:**
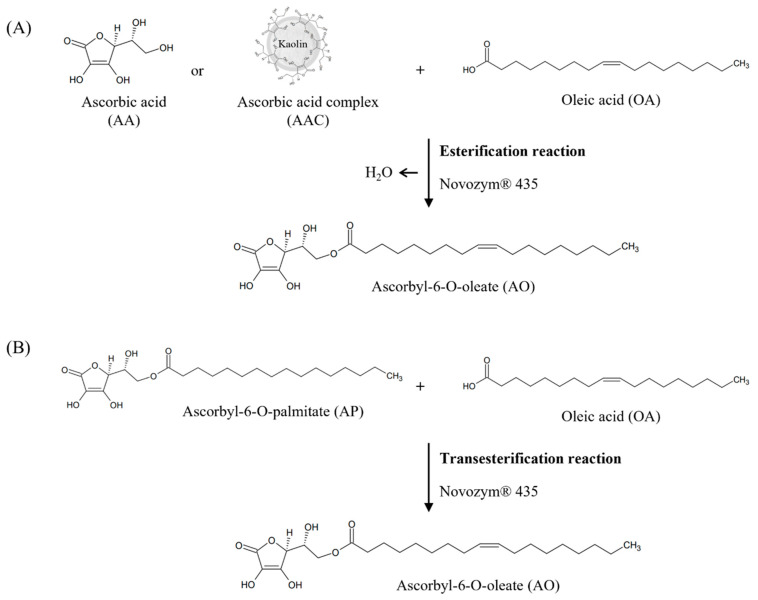
Ascorbyl-6-O-oleate synthesis schemes through lipase-catalysed esterification (**A**) and transesterification (**B**).

AO was synthesised via the lipase-catalysed esterification of AA and OA (AA–OA) at a 1:1 molar ratio, using Novozym^®^ 435 as the catalyst. In this reaction, AA (2.5 g, 14.5 mmol) and OA (4.1 g, 14.5 mmol) were combined (Figure 2). Novozym^®^ 435 was added immediately after adding AA, OA, the molecular sieve, and 15 mL acetone to a 250 mL screw flask; 10 wt% enzyme and a 10 wt% molecular sieve with respect to the substrate were used. The screw flask was filled with nitrogen and left in a shaking water bath at 50 °C and 200 rpm to react for 72 h.

#### 2.2.2. Synthesis of AO Using AA Complex

AO was synthesised via lipase-catalysed esterification of an ascorbic acid complex (AAC) and OA (AAC–OA) in a 1:1 mol ratio using Novozym^®^ 435 (Figure 2). AAC was prepared through a complexation by completely dissolving AA in methanol at 35 °C, adding an equal quantity of kaolin, and then concentrating the mixture under reduced pressure. AO was synthesised using AAC in 15 mL acetone or 15 mL hexane; the AO synthetic yields were compared between these two solvents. The quantities of the enzyme and molecular sieve and reaction conditions (e.g., temperature, reaction time, shaking speed) were the same as those employed for the AA-OA synthesis.

#### 2.2.3. Purification of AO Synthesised by Lipase-Catalysed Esterification

The enzyme and molecular sieve were filtered from the AA-OA reaction product, and the solvent was removed under reduced pressure from the remaining filtrate using a rotary evaporator. Subsequently, 120 mL hexane was added to the reaction product and mixed vigorously before centrifugation (2500 rpm, 10 min) and removal of the supernatant (unreacted OA). The product was washed two more times with 25 mL hexane. Subsequently, 10 mL ethyl acetate was added to the washed reaction product to extract AO, and the solvent was subsequently removed. To recover AO from the hexane used for washing (120 + 25 + 25 mL), a small amount of the hexane layer was purified by silica gel column chromatography. After packing the column with 5 g silica, 3.5 mL hexane layer was loaded into the column and approximately 75 mL hexane was passed through the column to remove OA. AO was eluted by adding a mixed solvent of ethyl acetate (EA), methanol, and water (volumetric ratio of 80:20:5).

To purify the AAC−OA reaction product synthesised in acetone, the enzyme and molecular sieve were removed using a filter paper, the solvent was removed from the remaining filtrate, and then the solution was purified via liquid–liquid extraction. For AA removal, 40 mL EA and 20 mL water were added to the reaction product filtrate and mixed vigorously before centrifugation (3500 rpm, 10 min) to separate the layers. After collecting the upper layer (EA layer) and removing the solvent, half of the solution was purified via hexane–acetonitrile (ACN) extraction, and the other half was purified through hexane–90% methanol (methanol:water = 9:1, *v*/*v*) extraction. For hexane–ACN extraction, 21 mL hexane and 7 mL ACN were added and left at 50 °C, followed by vigorous mixing and centrifugation (2500 rpm, 5 min) to separate the layers. After collecting the lower layer (ACN), extraction was repeated four times using 21 mL fresh hexane to fully remove OA. Finally, after complete removal of OA from the ACN layer, the solvent was removed, and 0.5 g AO was obtained. For hexane–90% methanol extraction, 12 mL hexane and 12 mL 90% methanol (methanol:water = 9:1, *v*/*v*) were used. The 90% methanol layer was recovered using the same method as that of the hexane–ACN extraction. The repeated extraction of OA was conducted using 12 mL hexane for purification. Finally, after removing OA from the 90% methanol layer, the solvent was removed, the resulting solution was freeze-dried for 12 h at −80 °C, and 0.5 g AO was obtained.

### 2.3. Synthesis and Purification of AO by Lipase-Catalysed Transesterification

#### 2.3.1. Lipase-Catalysed Transesterification Reaction for AO Synthesis

Mixtures of AP and OA were prepared in molar ratios of 1:1, 1:2, and 1:3 by combining 6.01 g of AP with 4.10 g, 8.19 g, and 12.29 g of OA, respectively, corresponding to 14.5 mmol of AP and 14.5, 29.0, and 43.5 mmol of OA. These mixtures were subjected to lipase-catalysed transesterification (AP-OA) using Novozym^®^ 435 to synthesise AO (Figure 2). The quantities of the enzyme and molecular sieve were each 10 wt% of the substrate. AP, OA, the molecular sieve, and acetone (5× the substrate) were added to a 250 mL screw flask. Before the reaction initiation, Novozym^®^ 435 was immediately added, and the screw flask was filled with nitrogen and left in a shaking water bath (50 °C, 200 rpm) to react for 72 h.

#### 2.3.2. Purification Process of AO Synthesized by Lipase-Catalysed Transesterification

The enzyme and molecular sieve were filtered from the AP–OA reaction product (molar ratio of 1:3), after which the solvent was removed from the remaining filtrate under reduced pressure using a rotary evaporator. AP was separated by dissolving the reaction product filtrate in 15 mL acetone, adding 450 mL hexane, and leaving the mixture at 4 °C. The resulting precipitate was removed, the hexane layer was left at −20 °C, and the resulting precipitate was removed again. The product precipitate was purified via hexane–ACN extraction; 150 mL ACN was added to the hexane layer with the precipitate removed and left at 50 °C before mixing vigorously, followed by centrifugation (3000 rpm, 5 min) and collection of the ACN layer, which contained dissolved AO. Subsequently, 450 mL fresh hexane was added, mixed, and centrifuged to remove the fatty acids (OA and palmitic acid (PA)). The supernatant was removed, and the product was washed 3–5 times with hexane. Finally, after the fatty acids were removed from the ACN layer, the solvent was removed, and 10 mL EA and 5 mL water were added to separate the layers. The EA layer was collected, and the solvent was removed again to obtain AO (3.1 g).

### 2.4. Analysis

#### 2.4.1. Thin Layer Chromatography (TLC)

TLC was performed to verify the reaction and purity of the product. EA:methanol:water = 80:20:5 (*v*/*v*/*v*) was used as the elution solvent. AA, AP, and AO were analysed under ultraviolet (UV) light at 254 nm. To analyse the fatty acids, the TLC plate was stained with 0.1% 2,7-dichlorofluorescein in 95% ethanol, and examined at a wavelength of 365 nm.

#### 2.4.2. High-Performance Liquid Chromatography–Evaporative Light Scattering Detection (HPLC-ELSD)

To verify the yield and purity of AO, we performed HPLC (Younglin SP930D, Anyang, Korea) with a YMC-Pack PVA-SIL-NP column (250 mm × 4.6 mm, D.S 5 µm, 12 nm, YMC Europe) and ELSD (ZAM300, Schambeck SFD GmbH, Germany). The temperature and pressure of the detector were set to 60 °C and 2.2 bar, respectively. For the mobile phase solvent, an isocratic elution (EA:methanol:water = 84:20:1) was used at a flow rate of 1 mL/min. The samples were dissolved at approximately 1 mg/mL in the mobile phase solvent. The sample volume was 20 μL, and the running time was 6 min. The synthesis yield was calculated using the following equation:

(1) $$\mathrm{Synthesis}\;\mathrm{yield}\;(\%)=\frac{\mathrm{area}\;\mathrm{of}\;\mathrm{AEs}}{\mathrm{area}\;\mathrm{of}\;\mathrm{AEs}+\mathrm{area}\;\mathrm{of}\;\mathrm{OA}}\;\times \;100$$

#### 2.4.3. Gas Chromatography–Flame Ionisation Detection (GC-FID)

To determine the of AP and AO ratios in the AEs synthesised by the transesterification reaction, AEs were isolated by TLC, methylated, and analysed via GC. A TLC plate (silica gel 60 F_254_, Glass, 20 × 20 cm, MERCK CO., Darmstadt, Germany) was used to isolate AEs with an elution solvent of EA:methanol:water = 80:20:5 (by volume), and scraped off only the isolated AE band. The substances separated via TLC were subjected to methylation to convert the fatty acyl groups of AEs to methyl esters. For methylation, 1.5 mL of 0.5 N NaOH (in methanol) was added, mixed for 30 s, reacted for 10 min at 85 °C, and frozen. Subsequently, 2 mL of 14% BF_3_ (in methanol) was added, mixed for 1 min, reacted for 10 min at 85 °C, and then froze again. Finally, 1 mL saturated NaCl solution and 2 mL iso-octane were added, mixed for 1 min, and centrifuged (2500 rpm, 3 min). The supernatant was passed through an anhydrous sodium sulfate column and collected in a GC vial. GC was performed using a gas chromatograph (Agilent, Santa Clara, USA) and an SP^TM^-2560 capillary column (biscyanopropyl polysiloxane, 100 m × 0.25 mm, 0.25 µm film thickness, Supelco, Bellefonte, PA, USA). The oven temperature was maintained at 100 °C for 4 min, increased at a rate of 3 °C/min to 240 °C, and then maintained for 17 min. The carrier gas flow rate was 1.0 mL/min with the split ratio of 200:1. The flame ionisation detector and injector temperatures were 285 °C and 225 °C, respectively, and the total analysis time was 70 min. Supelco 37 component FAME mix (Sigma-Aldrich) was used as the standard. The conversion rate from AP to AO was determined using the following equation:

(2) $$\mathrm{Conversion}\;\mathrm{rate}\;(\%)=\frac{\mathrm{area}\;\mathrm{of}\;\mathrm{methyl}\;\mathrm{oleate}}{\mathrm{area}\;\mathrm{of}\;\mathrm{methyl}\;\mathrm{oleate}+\mathrm{area}\;\mathrm{of}\;\mathrm{methyl}\;\mathrm{palmitate}}\;\times \;100$$

#### 2.4.4. ^1^H-NMR and ^13^C-NMR Spectroscopy

The AP and AO structure was verified through ^1^H-NMR and ^13^C-NMR analyses. After dissolving 30 mg sample in 700 μL DMSO-d6, the solution was added to a 600 MHz NMR tube for analysis. The chemical shift (ppm) for DMSO-d6 was δ = 2.50 and 39.98 for ^1^H and ^13^C, respectively. A Bruker Avance III-600 spectrometer (Bruker BioSpin, Billerica, MA, USA) was used with a Bruker Magnet (Bruker BioSpin). SpecMan software (Advanced Chemistry Development Inc., Toronto, ON, Canada) was used as the ^1^H-NMR and ^13^C-NMR analysis software.

#### 2.4.5. Fourier Transform Infrared Spectroscopy (FT-IR)

FT-IR analysis (Bruker Vertex 80v, Bruker Optics Inc., GmbH, Germany) was performed to verify the AP and AO structures. Approximately 5 mg of sample was placed on a horizontal attenuated total reflectance surface coated with diamond crystals. The spectral range was 400–4000 cm^−1^, and a deuterated triglycine sulfate detector and SiC source were used. The number of scans was 16.

#### 2.4.6. Liquid Chromatography Electrospray Ionisation Tandem Mass Spectrometry (LC-ESI-MS/MS)

For the LC-ESI-MS/MS, we used an Agilent 6470 Triple Quad LC Mass system fitted with an electrospray ionisation (ESI) source (Agilent, Palo Alto, CA, USA) and Mass Hunter Qualitative Analysis Software B.07.00 to verify the analysis results. The samples were dissolved in methanol and injected directly to the mass spectroscopy system at a volume of 10 μL. For the mobile phase, we used a 50:50 ratio of water with 0.1% formic acid and ACN with 0.1% formic acid in the isocratic mode at a flow rate of 0.5 mL/min. We used N_2_ gas for the fragmentation of precursor ions. The analysis conditions are summarised in Table 1.

#### 2.4.7. Differential Scanning Calorimetry (DSC)

DSC (Mettler-Toledo GmbH, Schwerzenbach, Switzerland) was conducted to confirm the melting behaviour of AP and AO. A total of 1–5 mg of the sample was placed in an aluminium (Al) pan, which was sealed with an Al lid. To obtain melting profiles, AP was heated from 0 °C to 200 °C and AO was heated from −25 °C to 200 °C, both at a rate of 5 °C/min.

## 3. Results

### 3.1. Synthesis of AO by Lipase-Catalysed Esterification

To verify whether the AAC helped increase AO yields, we compared the yields in acetone when using AA-OA (AA and OA) or AAC-OA (AAC and OA) as the substrates (Table 2). We collected samples every 24 h during the 72 h reaction time and analysed these samples through HPLC. After 24 h of reaction, the AO yield of AAC-OA was lower (13.8%) than that of AA-OA (18.3%). When the reaction was concluded at 72 h, both AA-OA and AAC-OA showed yields of 19.7%, with no significant difference between the two substrates (*p* < 0.05). Thus, synthesis using the AAC exhibited a lower initial synthesis rate, suggesting that the dispersion of the substrate did not significantly increase the AO yield. Additionally, when AAC-OA was reacted in hexane, in which AA is insoluble, AO was not synthesised. This suggested that the low solubility of AA in the reaction solvent is a limiting factor for AO synthesis.

### 3.2. Purification of AO Reaction Product Synthesised by Lipase-Catalysed Esterification

**Figure 3:**
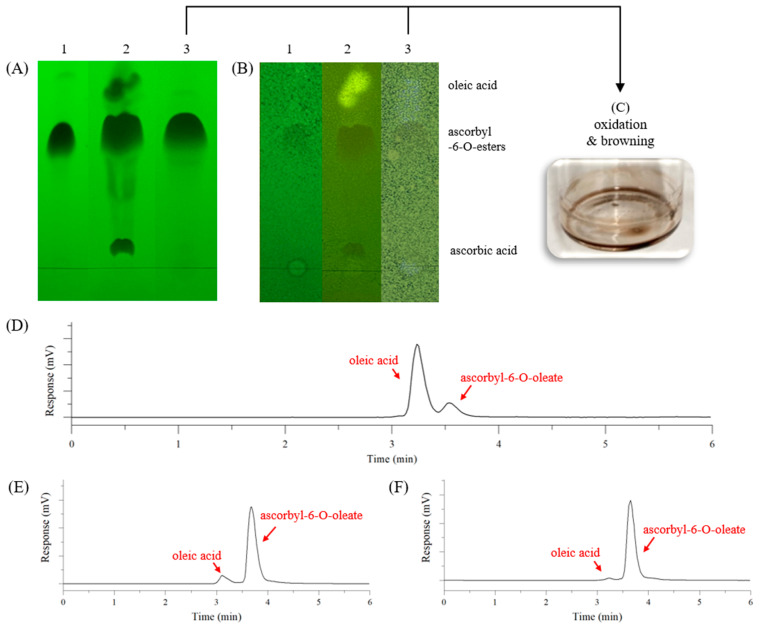
Purification of the reaction product synthesised through lipase-catalysed esterification. (**A**) Thin layer chromatography (TLC) of ascorbic acid (AA) and ascorbyl-6-O-oleate (AO) observed at 254 nm. (**B**) TLC of fatty acid observed at 365 nm. 1, AO obtained from hexane washing followed by EA extraction; 2, AO dissolved in hexane by hexane washing; 3, AO obtained after silica gel column chromatography (ethyl acetate: methanol: water = 80:20:5 (*v*/*v*/*v*)). (**C**) Oxidation and browning of AO eluted from silica gel column chromatography. (**D**) HPLC-ELSD chromatogram of esterification reaction product of ascorbic acid complex (AAC) and OA. (**E**) HPLC-ELSD chromatogram of AO, which finally purified the esterification reaction product of AAC and OA with hexane and acetonitrile. (**F**) HPLC-ELSD chromatogram of AO, which finally purified the esterification reaction product of AAC and OA with hexane and 90% methanol.

When the AA-OA reaction product filtrate was purified by hexane washing followed by EA extraction, we successfully verified via TLC that high-purity AO was obtained (Lane 1 of Figure 3A), but the final purification yield was <1%. Lane 3 in Figure 3A shows the AO obtained by purifying the composition of Lane 2 using silica gel column chromatography. Although the same band shape was observed as in Lane 1 in Figure 3A, which achieved high purity, AO oxidised as soon as it was loaded into the silica gel column, and a brown substance was eluted (Figure 3C).

When we mixed EA (2 vol) and water (1 vol) into the filtrate of the reaction product of AAC-OC in acetone (Figure 3D), water-soluble AA was present in the lower water layer, while lipid-soluble OA and amphiphilic AO were present in the upper EA layer. After removing EA by evaporation and adding hexane (3 vol.) and ACN (1 vol.), OA was extracted in the hexane (upper) layer and AO was again present in the ACN (lower) layer. When we analysed AO obtained by this method, the HPLC results show 94.8 area% for AO and 5.2 area% for OA (Figure 3E).

When we used 90% methanol (methanol: water, 9:1, *v*/*v*), it successfully replaced ACN and enabled the purification of AO. The HPLC results show 97.2 area% for AO and 2.8 area% for OA, respectively (Figure 3F).

### 3.3. Synthesis of AO by Lipase-Catalysed Transesterification

AO was synthesised using AP:OA molar ratios in the range of 1:1–1:3 and compared the conversion rates (Table 3). AP and AO were both present in the AEs isolated by TLC, and we performed the GC of methyl palmitate and methyl oleate converted from these substances to determine the amount of AP converted to AO. After reacting for 72 h, the conversion rates ranged from 50.1% (1:1) to 73.8% (1:3). In all cases, the conversion rates gradually increased up to 48 h, after which there was no significant change. Under the same conditions (1:1 molar ratio, in acetone at 50 °C), the yield of AO by esterification was 19.7% (Table 2); however, the conversion rate by transesterification was 50.1%, indicating that approximately twice as much AO was synthesised. Furthermore, when the molar ratio was increased to 1:3, over three times as much AO (73.8%) was synthesised.

### 3.4. Purification of AO by Lipase-Catalysed Transesterification

**Figure 4:**
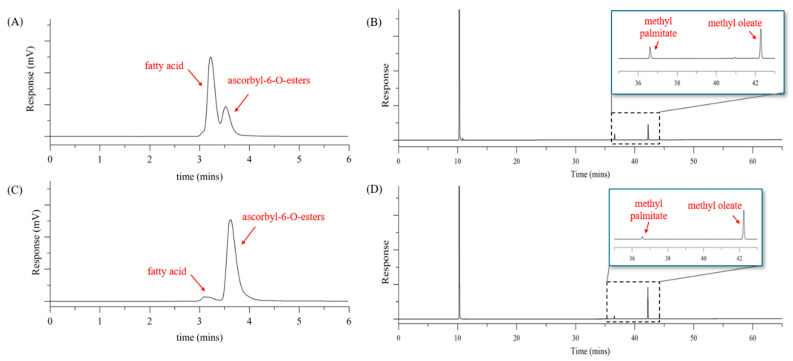
HPLC-ELSD and GC-FID chromatograms of the transesterification reaction product of ascorbyl-6-O-palmitate and oleic acid (molar ratio of 1:3) before and after purification. (**A**) HPLC-ELSD chromatogram of transesterification reaction product. (**B**) GC-FID chromatogram of transesterification reaction product. (**C**) HPLC-ELSD chromatogram of ascorbyl-6-O-esters, which finally purified. (**D**) GC-FID chromatogram of ascorbyl-6-O-esters, which finally purified.

To purify the highest-yield transesterification reaction product (AP:OA = 1:3, molar ratio), the filtrate was completely dissolved in acetone, and hexane was used to recrystallise AP sequentially at temperatures of 4 °C and −20 °C. To prevent separation difficulties due to the formation of the oleo gel, a sufficient quantity of hexane is required. The remaining solution after re-crystallisation contained dissolved fatty acids (OA, PA) and the AE. In the hexane layer after re-crystallisation at 4 °C, the ratios of AO and AP comprising the AE were 90.5% and 9.5%, respectively. When the 4 °C hexane layer was re-crystallised at −20 °C, the ratio of AO in the hexane layer did not change significantly (91.0%) (Table 4 and Figure 4).

Thus, when re-crystallisation was performed at 4 °C, most of the AP could be separated from AO, and AP was not further precipitated at −20 °C. ACN (1 vol.) was added to the remaining solution (3 vol.) after re-crystallisation, and the AE (AP:AO = 91%:9%) was extracted with ACN, similar to that achieved through the purification method for the esterification reaction product. Thereafter, fresh hexane was added serially (3–5 times) to remove the fatty acids remaining in ACN. After the evaporation of ACN, the solution was separated into EA (2 vol.) and water (1 vol.) layers, and the AE was present in the EA (upper) layer. When we analysed the AE obtained after EA evaporation, HPLC results showed 94.3 area% for the AE and 5.7 area% for fatty acids (Figure 4).

### 3.5. AO Analysis

#### 3.5.1. ^1^H-NMR Spectroscopy

**Figure 5:**
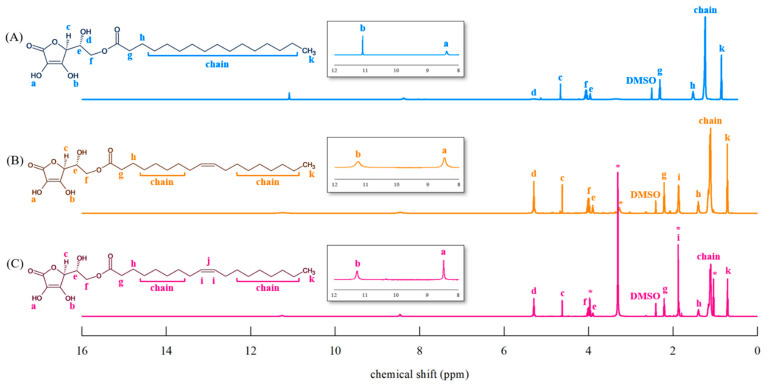
^1^H-NMR spectra of ascorbyl-6-O-palmitate (AP) and ascorbyl-6-O-oleate (AO). (**A**) AP, (**B**) AO obtained by finally purifying the esterification reaction product of AAC and OA with hexane and 90% methanol, (**C**) AO obtained by finally purifying the transesterification reaction product of AP and OA with ethyl acetate and water. The detailed purification procedures of AO are described in Figure 1. The solvent peak is marked *. The various colored letters (a–k) indicated in each structure are represented in the ^1^H-NMR spectrum with letters of the same color corresponding to the respective chemical shifts.

Figure 5 shows the ^1^H-NMR spectra of the standard AP (Figure 5A) and AO obtained in this study (Figure 5B,C).

AP ^1^H-NMR (DMSO-d6, 600 MHz); δ (ppm) 11.10 (br, 1H, –OH), 8.41(br, 1H, –OH), 5.28 (br, 1H, –OH), 4.67 (d, 1H, –CH), 4.10–4.04 (m, 2H, CH_2_–O), 3.97–3.95 (m, 1H, –CH–OH), 2.33–2.30 (t, 2H, –CH_2_CO), 1.53 (m, 2H, –CH_2_–), 1.24–1.31 (m, 24H, –CH_2_–), 0.86 (t, 3H, –CH_3_).

AO (AO obtained by purifying AAC–OA reacted in acetone with hexane and 90% methanol) ^1^H-NMR (DMSO-d6, 600 MHz); δ (ppm) 11.10 (br, 1H, –OH), 8.41 (br, 1H, –OH), 5.32 (m, 2H, CH=CH), 4.67 (d, 1H, –CH), 4.10–4.04 (m, 2H, CH_2_–O), 3.97–3.95(m, 1H, –CH–OH), 2.33–2.30 (t, 2H, –CH_2_CO), 1.97–2.04 (m, 4H, CH_2_CH=CHCH_2_), 1.53 (m, 2H, –CH_2_–), 1.24–1.31 (m, 20H, –CH_2_–), 0.86 (t, 3H, –CH_3_).

AO (AO obtained by purifying the AP–OA reaction product reacted in a 1:3 molar ratio) ^1^H-NMR (DMSO-d6, 600 MHz); δ (ppm) 11.10 (br, 1H, –OH), 8.41 (br, 1H, –OH), 5.32 (m, 2H, CH=CH), 4.67 (d, 1H, –CH), 4.10–4.03 (m, 2H, CH_2_–O), 3.97–3.95 (m, 1H, –CH–OH), 2.33–2.30 (t, 2H, –CH_2_CO), 1.97–2.04 (m, 4H, CH_2_CH=CHCH_2_), 1.53 (m, 2H, –CH_2_–), 1.24–1.31 (m, 20H, –CH_2_–), 0.86 (t, 3H, –CH_3_).

#### 3.5.2. ^13^C-NMR Spectroscopy

**Figure 6:**
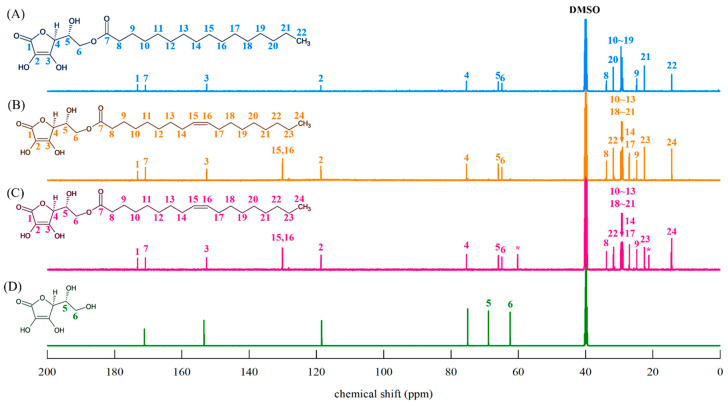
^13^C-NMR spectra of ascorbyl-6-O-palmitate (AP), and ascorbyl-6-O-oleate (AO). (**A**) AP, (**B**) AO obtained by finally purifying the esterification reaction product of AAC and OA with hexane and 90% methanol, (**C**) AO obtained by finally purifying the transesterification reaction product of AP and OA with ethyl acetate and water, (**D**) ascorbic acid. The detailed purification procedures of AO are described in Figure 1. The solvent peak is marked *. The various colored numbers (1–24) indicated in each structure are represented in the ^13^C-NMR spectrum with numbers of the same color corresponding to the respective chemical shifts.

Figure 6 shows the ^13^C-NMR spectra of standard AP (Figure 6A) and AO obtained in this study (Figure 6B,C).

AP ^13^C-NMR (DMSO-d6, 600 MHz); δ (ppm) 173.16 (C-1), 170.80 (C-7), 152.80 (C-3), 118.66 (C-2), 75.45 (C-4), 65.94 (C-5), 64.88 (C-6), 33.85 (C-8), 31.76 (C-20), 29.51–28.93 (C-10–19), 24.82 (C-9), 22.55 (C-21), 14.38 (C-22).

AO (AO obtained by purifying AAC–OA reacted in acetone with hexane and 90% methanol) ^13^C-NMR (DMSO-d6, 600 MHz); δ (ppm) 173.13 (C-1), 170.80 (C-7), 152.61 (C-3), 130.07 (C-15 or C-16), 130.04 (C-15 or C-16), 118.65 (C-2), 75.45 (C-4), 65.94 (C-5), 64.88 (C-6), 33.82 (C-8), 31.75 (C-22), 29.56–28.95 (C-10–13, C-18–21), 27.03 (C-14, C-17), 24.82 (C-9), 22.55 (C-23), 14.38 (C-24).

AO (AO obtained by purifying the AP–OA reaction product reacted in a 1:3 molar ratio) ^13^C-NMR (DMSO-d6, 600 MHz); δ (ppm) 173.18 (C-1), 170.81 (C-7), 152.62 (C-3), 130.10 (C-15 or C-16), 130.06 (C-15 or C-16), 118.64 (C-2), 75.46 (C-4), 65.94 (C-5), 64.88 (C-6), 33.82 (C-8), 31.73 (C-22), 29.54–28.92 (C-10–13, C-18–21), 27.02 (C-14, C-17), 24.81 (C-9), 22.54 (C-23), 14.39 (C-24).

#### 3.5.3. FT-IR Spectroscopy and LC-ESI-MS/MS

**Figure 7:**
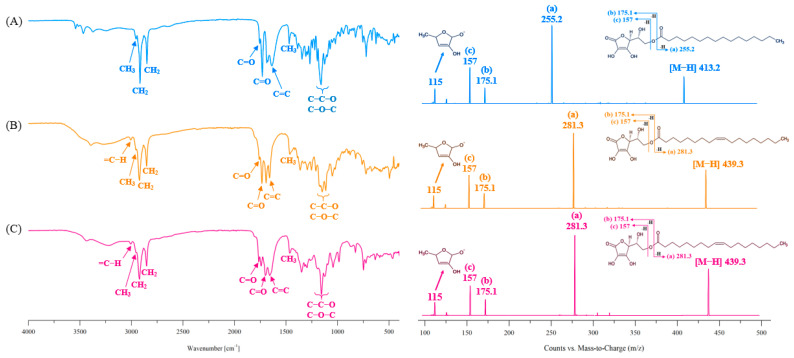
FT-IR (left) and LC-ESI-MS/MS (right) spectra of ascorbyl-6-O-palmitate (AP) and ascorbyl-6-O-oleate (AO). (**A**) AP, (**B**) AO obtained by finally purifying the esterification reaction product of AAC and OA with hexane and 90% methanol, (**C**) AO obtained by finally purifying the transesterification reaction product of AP and OA with ethyl acetate and water. In the LC-ESI-MS/MS spectra, the various colored letters (a–c) indicated in each structure are represented by letters of the same color in the corresponding ion spectra.

Figure 7 shows the FT-IR and LC-ESI-MS/MS spectra of standard AP (Figure 7A) and AO obtained in this study (Figure 7B,C). In the FT-IR spectrum, peaks are observed at 2956 cm^−1^ corresponding to CH_3_ stretching, 2921 and 2852 cm^−1^ corresponding to CH_2_ stretching, 1733.3 cm^−1^ corresponding to C=O stretching of the ester group, 1758 cm^−1^ corresponding to the C=O stretching in AA, 1660 cm^−1^ corresponding to C=C stretching in AA, 1461 cm^−1^ corresponding to bending of CH_3_ at the end of the acyl group, and 1100–1190 cm^−1^ corresponding to C–C–O stretching of the ester and C–O–C of AA [1,26].

In the LC-ESI-MS/MS spectra (Figure 7), a molecular ion [M–H]^−^ peak is observed at *m/z* 413.2 owing to the fact that the molecular weight of standard AP is 414 g/mol, which is verified as a fragmented ion peak by MS/MS. [AA–H_2_O]^−^, [AA–H]^−^, and [PA–H]^−^ exhibit peaks at *m/z* 157, 175, and 256, respectively, owing to the cleavage of the ester bonds between AA (molecular weight = 176.12) and PA (molecular weight = 256.43) (Figure 7A). The *m/z* 115 peak indicates the presence of C_5_H_7_O_3_^−^ owing to the deprotonation of fragmented AA (Figure 7A). [AA–H_2_O]^−^, [AA–H]^−^, and [OA–H]^−^ depict peaks at *m/z* 157, 175, and 282, respectively, ascribed to the cleavage of the ester bonds between AA (molecular weight = 176.12) and OA (molecular weight = 282.46), and the *m/z* 115 peak confirms the presence of C_5_H_7_O_3_^−^ due to the deprotonation of fragmented AA (Figure 7B,C).

#### 3.5.4. DSC

**Figure 8:**
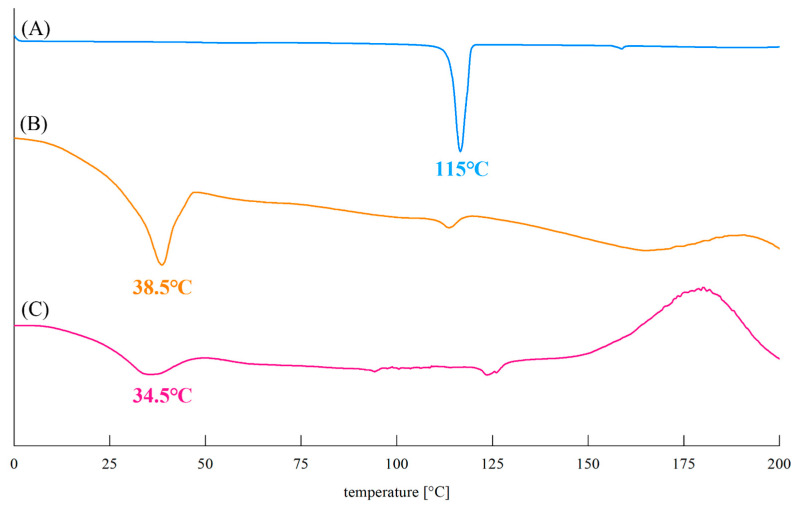
Differential scanning calorimetry (DSC) thermograms of ascorbyl-6-O-palmitate (AP) and ascorbyl-6-O-oleate (AO). (**A**) AP, (**B**) AO obtained by finally purifying the esterification reaction product of AAC and OA with hexane and 90% methanol, (**C**) AO obtained by finally purifying the transesterification reaction product of AP and OA with ethyl acetate and water.

Figure 8 shows the DSC thermograms for standard AP (Figure 8A) and AO obtained in this study (Figure 8B,C). The thermogram of standard AP exhibits a dissolution peak at 113.80–119.24 °C (Δ_fus_H = 138.1 J/g), whereas that of AO has a broad dissolution peak at 23.1–46.7 °C (Δ_fus_H = 51.8, 20.5 J/g).

## 4. Discussion

This study investigated the reaction solvents for the synthesis of ascorbyl-6-O-oleate and presented a method for purifying AO from the reactants. Additionally, the synthesis and purification methods for AO through transesterification enzyme reactions were analysed, along with structural characterisation, to confirm the formation of AO products.

### 4.1. Reaction Solvents for AO Synthesis

Reaction conditions such as temperature, enzyme type and concentration, reaction solvent, and acyl donor type must be considered for the enzymatic synthesis of AEs [15,16,17,18,19,20,21,22,23,24]. Holtheuer et al. demonstrated that Novozym^®^ 435 exhibited a higher initial reaction rate, yield, and productivity than Amano Lipase PS, Novozym^®^ TLIM, Novozym^®^ Novo 40086, or Novozym^®^ RMIM, making it the most suitable enzyme for AE synthesis [18]. Among the four hydroxyl groups in AA, the C6 hydroxyl group has the highest accessibility and reactivity; therefore, this hydroxyl group reacts selectively with the carboxyl group in OA to promote the esterification reaction [27].

It is essential to choose an organic reaction solvent with appropriate polarity to simultaneously dissolve the substrates, AA and OA. While AA is highly soluble in polar solvents, OA is highly soluble in nonpolar solvents, making the choice of solvents very important. Moreover, the polarity of a solvent can have a significant effect on enzyme activity, and affects the extent of control of the essential water layer surrounding the enzymes [28]. Therefore, to predict enzyme activity, logP (octanol–water partition coefficient) was used to represent the polarity of the organic solvent. Previous studies on the esterification synthesis of AEs have demonstrated high yields using t-butanol (logP = 0.80) or t-amyl alcohol (logP = 1.31) as the reaction solvent, whereas yields were lower when using acetone (logP = −0.23) [9,15,19]. Although several explanations can be surmised for the differences in yield, acetone has a relatively high polarity and will readily absorb water needed to sustain enzymatic activity [29]. Additionally, the low solubility of substrate AA in acetone may hinder its participation in the reaction.

However, Sanofi’s solvent selection guide categorises organic solvents into one of four grades from “recommended” to “banned”, classifying t-butanol and t-amyl alcohol as “substitution advisable”. Moreover, acetone is classified as “recommended”, and can be used as a reaction solvent because it is inexpensive and safe. Therefore, it could be utilised as an economical industrial solvent for the development of enzymatic processes for producing AO [14,25]. Additionally, acetone is recognised as a permissible solvent in the Korean Food Additives Code, making it more suitable for industrial applications. To increase the lower yield of AO compare to that of other organic solvents [9], there have been attempts at syntheses using the AAC by attaching AA to kaolin, rather than using AA directly. The purpose of using the AAC is to enhance the dispersion of the substrate, resulting in a broader contact area between the substrate and enzyme, thereby overcoming the low solubility of AA in acetone. However, the results show that AAC did not significantly enhance the AO yield compared to using AA directly (Table 2).

### 4.2. Purification of AO from Reactants

To date, previous studies on the purification of AO synthesised by the enzymatic reaction have mostly conducted preparative HPLC to elute AO and isolate high-purity AO. However, this is an expensive, time-consuming process, making it unsuitable for mass production [4,14,29]. Other purification methods leveraged fatty acids’ high solubility in hexane, performing multiple washing steps to remove unreacted fatty acids from the AO product [15]. Alternatively, AO was extracted using EA [26] or diethyl ether (DE) [16], while water was used to extract AA. However, DE incurs safety concerns owing to its low boiling and flash points, restricting its industrial use [25]. Therefore, we used hexane and EA, which are listed in the Korean Food Additives Code and permitted for use in the manufacture of food ingredients, to obtain high-purity AO from the AA-OA reaction product (Figure 3). However, purification using EA extraction after hexane washing had a very low yield. The low purification yield was because most of synthesised AO dissolved in hexane was used for washing, as shown in Lane 2 of Figure 3A. Thus, not only can substrate OA act as a solvent [30] but AO also cannot be efficiently precipitated in hexane because it has a low melting point owing to its oleoyl group [31]. Additionally, purification using a silica gel column was attempted, but discoloration occurred immediately upon loading, making it unsuitable for purification. Silica gel can act as an oxidation catalyst [16,32], and when AA is oxidised, it is converted to dehydroascorbic acid, which is subsequently hydrolysed in the presence of water to form substances such as 2,3-diketogluonic acid that cause discolouration [33]. This is consistent with previous studies that attempted preparative HPLC purification using a silica column but reported colour change due to the oxidisation of AO [15].

The low melting point (i.e., limited crystallisation) and readily oxidisation of AO made its purification challenging. To overcome these difficulties, we attempted liquid–liquid extraction to separate the substances based on their solubility, while inhibiting the dissolution of AO due to the solvent effect of OA. According to Hoerr et al., OA dissolved very easily in organic solvents such as hexane, DE, acetone, methanol, and EA but showed low solubility in ACN (9.1 g/100 g ACN at 20 °C) [34]. Because hexane and ACN are immiscible, we predicted that they would separate cleanly into layers, with OA dissolved in hexane and AO dissolved in the ACN layer.

While ACN and methanol are both polar solvents, methanol is favoured over ACN based on solvent greenness [35] and is listed in the Korean Food Additives Code, indicating that it is permitted for unrestricted use in the food industry. However, methanol is a protic solvent with a hydroxyl group, and forms intermolecular hydrogen bonds. As a result, OA dissolves in methanol because the hydrogen atoms in the carboxyl group of OA compete with the hydrogen bonds between methanol molecules [34]. Conversely, ACN (dipole moment = 3.92, polarity index = 5.8) is an aprotic solvent that does not provide hydrogen atoms, and it is more polar, with a stronger dipole moment than methanol (dipole moment = 1.70, polarity index = 5.1) [36,37]. The hydrogen atoms in the carboxyl group of OA are unable to disrupt the highly polar field of ACN (due to nitrogen); thus, OA shows low solubility in ACN, making liquid–liquid extraction using hexane and ACN possible.

If ACN was replaced with methanol, this could be suggested as a more environmentally friendly purification method. However, because methanol can dissolve OA, it is difficult to use it for AO isolation. To overcome this limitation, we attempted to replace ACN with a mixture of methanol (polarity index = 5.1) and water (polarity index = 10.2) to increase the polarity. However, layer separation was difficult unless a suitable ratio was used, due to the formation of an oleo gel or a semi-solid layer at the interface. Interestingly, when 90% methanol (methanol:water, 9:1, *v*/*v*) was used, OA was present in the upper hexane layer and AO was present in the lower 90% methanol layer, allowing for readily layer separation. The 90% methanol was able to replace ACN, achieving an AO of 97.2 area% according to HPLC analysis (shown in Figure 3F). Consequently, when 2.05 g (14.5 mmol) of AA and 4.1 g (14.5 mmol) of OA were reacted, 1.32 g of AO was obtained, and after purification, 1 g of AO was obtained.

### 4.3. Synthesis of AO Through Transesterification Reaction

Acidolysis is a type of transesterification reaction involving the exchange of acyl groups between an ester and a fatty acid. To date, when producing AEs by lipase-catalysed transesterification of AA, fatty acid esters (methyl, ethyl, vinyl) or triacylglycerol in the ester form were used as acyl donors [4,14,21,22,23]. In this study, when we synthesised AO using an esterification reaction, the low solubility of AA in acetone inhibited AO synthesis; thus, we attempted acidolysis (i.e., transesterification) using AP in the ester form and OA as an acyl donor. As a result of attempting to synthesise AO through a transesterification reaction, when the substrate ratio of AP and OA was 1:3, the conversion rate was more than three times that of the esterification reaction (Table 3). This high conversion rate is possible because, unlike the low solubility of AA in acetone, AP is highly soluble (6 g/50 mL in acetone at 50 °C). Thus, the transesterification reaction using AP as a substrate in acetone, a green solvent recommended for environmentally friendly industrial use, could be a good approach to increase AO yields.

Because AP was used as the substrate, it is necessary to separate AP and AO for purification of the transesterification reaction product. Generally, AEs have low solubility in hexane. However, as mentioned above, excessive quantities of OA could act as a solvent, causing AO to dissolve in hexane. In addition, because AP has a higher melting point than AO, we predicted that AO would dissolve while AP would be precipitated in the presence of hexane and excess OA at a specific low temperature. Using this method, when recrystallising sequentially in hexane at 4 °C and −20 °C, the ratio of AO and AP constituting AE in the hexane layer after recrystallisation was 91% and 9%, respectively (Table 4). Following this, the liquid–liquid extraction was performed sequentially using hexane–ACN followed by EA–water, resulting in 94.3 area% for AE (shown in Table 4). Consequently, when 6.01 g (14.5 mmol) of AP and 12.29 g (43.5 mmol) of OA were reacted, 4.7 g of AO was obtained, and after purification, 3.1 g of AE was obtained.

### 4.4. Structural Identification and Analysis of AO

AO obtained by purifying AAC–OA reaction in acetone with hexane and 90% methanol, as well as AO obtained from the AP–OA reaction product in a 1:3 molar ratio, were analysed using ^1^H-NMR, ^13^C-NMR, FT-IR, LC-ESI-MS/MS, and DSC. Since AO is not commercially available as a standard, AP was used as the standard instead.

AO is composed of AA, which features a unique 1-one-2,3-diol-2-ene system that contributes to its antioxidant properties, instability, and poor physicochemical characteristics. During oxidation, the 3-OH group (pKa = 4.17) ionises first, forming a 3-monoanion (pKa 11.57), and upon losing an electron, a radical species is generated (pKa −0.86). The ionisation of the 2-OH group in the radical leads to further electron loss, resulting in the formation of inactive dehydroascorbic acid, making the ionisation of 3-OH a critical factor in the oxidation of AA [7]. Because 2-OH and 3-OH are essential structures for antioxidative properties, we confirmed the presence of peaks for these OH groups at 11.10 (b) and 8.41 (a) ppm through ^1^H-NMR [38]. We confirmed that the area ratios of the terminal CH_3_ peak (0.86 ppm) and the C-3 hydroxyl peak (8.41 ppm) were the same, at 1:0.26, in both standard AP and AO obtained in our study. The spectrum of ^1^H-NMR showed that AO obtained in our study was not oxidised. The i peaks (1.97–2.04 ppm) corresponding to the CH_2_ groups on either side of the olefin structure overlap with the EA peak in Figure 5C, which can also be observed in the ^13^C-NMR results below.

In the ^13^C-NMR spectrum (Figure 6), the presence of the 170.80 ppm (7) peak, corresponding to the ester bond, along with 130.04 ppm (15,16) peak representing the olefin structure of OA, confirms that AO was synthesised through esterification and transesterification. In previous studies, the presence of ester bonds at the C-6 hydroxyl group of AA resulted in an upfield shift of the C-5 signal and a downfield shift in the C-6 signal [39,40]. We observed a shift in the C-5 signal of AP and AO from 68.86 to 65.94 ppm and a shift from 62.43 to 64.88 ppm of the C-6 signal (shown in Figure 6), demonstrating that C-6 on AA was selectively esterified by OA. The EA peaks shown in the ^1^H-NMR spectra (Figure 5C) were identified at 60.22, 21.20, and 14.53 ppm in the ^13^C-NMR spectra (Figure 6C).

In the FT-IR spectrum, the AO obtained from esterification and transesterification reactions showed the same peaks at the 3006 cm^−1^ peak as standard AP. The 3006 cm^−1^ peak was observed only in the FT-IR spectrum of AO due to the =C–H stretching of OA [41]. The 1733.3 cm^−1^ peak, representing ester bonds, and 3006 cm^−1^ peak, representing the =C-O group in the olefin structure of OA, indicated the esterification of AA and OA, while the 1708 cm^−1^ peak, representing the carboxyl C=O of OA, was not observed [42].

In the LC-ESI-MS/MS spectrum of AO (molecular weight = 440 g/mol) obtained from the esterification and transesterification reactions, the fragmented ion peak for the molecular ion [M-H]^-^ peak at *m/z* 439.3 showed the same pattern as that of AP (shown in Figure 7).

The DSC results (Figure 8) for AO obtained in this study were significantly different than previously reported values, but the previously reported peaks vary greatly, including those at 56.1 °C (Δ_fus_H = 40.4 J/g), 62.4 °C (Δ_fus_H = 34.8 J/g), 68–72 °C (enthalpy of fusion not shown), and 83–84 °C (Δ_fus_H = 20–30 J/g) [15,16,26,43]. There are several possible explanations for the diverse reported melting points (enthalpy of fusion) for AO.

First, fatty acids and similarly structured molecules are polymorphic, indicating that they exist in several crystalline forms including hexagonal (α_H_), orthorhombic (β_O_), triclinic (β_T_), and monoclinic (β_M_) phases. Perini et al. reported that the crystal structure of AO can differ depending on purification and predicted that a less crystalline structure would be associated with a lower entropy of fusion (ΔfusS = ΔfusH/Tfus) [26]. The entropy of fusion of AO obtained in this study was 73.2 and 29.4 J/mol·K, and those in previous studies varied among 30.9, 45.6, and 53.5 J/mol·K [15,26,43], demonstrating that the final purification stage was different in all these studies. Thus, this difference is due to polymorphism.

Second, we can consider the effects of trace amounts of OA or AA remaining in AO. PA reduces the crystallinity of AP because it easily mixes with AP [44]. Given the similarities between AO and AP, the different crystallinities of AO could be ascribed to the presence of trace amounts of AA or OA that were not removed during purification.

Finally, the melting point of AO has been mentioned in several studies. In the ^1^H-NMR spectra, the C-2 and C-3 hydroxyl group peaks at 11.10 and 8.41 ppm are difficult to confirm; however, we can hypothesise their presence considering AO in a deprotonation state. In particular, as the deprotonation of the C-2 and C-3 hydroxyl groups on AO occurs during the process of oxidation, the likelihood of promoting oxidation is increased. AA is oxidised to dehydroascorbic acid, which has a melting point of more than 30 °C, suggesting the increased melting point with the oxidation of AO.

Thus, although we have suggested several possibilities to explain the diverse melting points and enthalpy of fusion for AO in this and previous studies, the exact reason is yet to be elucidated.

## 5. Conclusions

In this study, we synthesised and purified AO via lipase-catalysed esterification and transesterification. We presented an economical and ecofriendly synthesis method that can increase AO yield using acetone as the reaction solvent. Additionally, we presented a purification method that can replace preparative HPLC. The structure and characteristics of AO obtained by these methods were investigated in depth.

The esterification product can be purified through a multi-step process that typically involves solvent extraction, washing with appropriate solvents to remove impurities. For enhanced purity, the product may also be subjected to re-crystallisation and liquid–liquid extraction depending on the nature of the ester and the impurities present. In this study, an enzymatic esterification reaction was performed using the water-soluble substrate AA and the oil-soluble substrate OA, with acetone as the solvent capable of dissolving both substrates. However, the synthesis rate of AO was found to be over 2.5 times lower compared to that achieved through the enzymatic transesterification reaction conducted under the same conditions (1:1 molar ratio, 50 °C, acetone). Furthermore, when the molar ratio of AP to OA was increased to 1:3, a synthesis rate of over 73.8% was achieved, which is more than three times higher. Through this approach, this study proposed a process involving the sequential application of recrystallisation and liquid–liquid extraction on the AP-OA (1:3 molar ratio) reaction product, resulting in the highest yield of AO relative to the acyl donor used. Therefore, the transesterification reaction and purification method utilising AP as a substrate proposed in this study are believed to contribute to the commercialisation of AO synthesis.

Owing to its amphiphilic and antioxidative activity, we expect the applicability of AO in producing nanocarriers, such as micelles or liposomes, via self-assembly, while simultaneously performing antioxidative functions. As AO is readily ionised at a physiological pH, it may also improve interface properties and the efficiency of drug-delivery systems. The properties of AO are important motivators for its future application studies. In particular, AO could be used commercially in food products and other systems, such as cosmetics or pharmaceuticals. Thus, the application scope of AO needs to be explored further. Our findings provide basic data to determine the optimal conditions for the commercial synthesis of AO, design environmentally friendly purification methods, and explore potential uses of AO.

## Figures and Tables

**Table 1:** LC-ESI-MS/MS operating conditions.

Ion Source Type	ESI, Negative Ion Mode		
Gas temp	300 °C		
Gas flow rate	9 L/min		
Nebulizer gas	45 psi		
Sheath gas temp	320 °C		
Sheath gas flow	11 L/min		
Scan Type	Product ion (MS^2^ Scan range: *m/z* 100~500)		
	Compound	Fragment (V)	CE (V)
	Ascorbyl-6-O-palmitate	220	20
	Ascorbyl-6-O-oleate	170	20

**Table 2:** Synthesis yields of ascorbyl-6-O-oleate in esterification reaction products at different organic media according to reaction time.

Reaction Time (h)	Synthesis Yields (%) of Ascorbyl-6-O-Oleate		
	AA-OA ^(1)^	AAC-OA ^(2)^	
	Acetone	Acetone	Hexane
24	18.3 ± 0.1 ^b (3)^	13.8 ± 0.5 ^c^	- ^(4)^
48	19.2 ± 0.1 ^ab^	19.3 ± 0.5 ^ab^	-
72	19.7 ± 0.1 ^a^	19.7 ± 0.1 ^a^	-

^(1)^ AA-OA = lipase-catalysed esterification reaction of oleic acid (OA) with ascorbic acid (AA). ^(2)^ AAC-OA = lipase-catalysed esterification reaction of oleic acid (OA) with an ascorbic acid complex (AAC, ascorbic acid adsorbed on kaolin). ^(3) a–c^ Mean values with different letters above the quantitative values are significantly different (*p* < 0.05) according to Duncan’s multiple range test (mean ± standard deviation, *n* = 2). ^(4)^ - not detected.

**Table 3:** Conversion rates of ascorbyl-6-O-oleate in transesterification reaction products at different molar ratios according to reaction time.

Reaction Time (h)	Conversion Rates (%) of Ascorbyl-6-O-Oleate		
	Ascorbyl-6-O-Palmitate: Oleic Acid		
	1:1	1:2	1:3
24	38.6 ± 0.1 ^f^	57.2 ± 0.1 ^d^	69.8 ± 0.1 ^b^
48	49.0 ± 0.1 ^e^	64.9 ± 0.1 ^c^	73.2 ± 0.1 ^a^
72	50.1 ± 0.1 ^e^	64.8 ± 0.1 ^c^	73.8 ± 0.1 ^a^

^a–f^ Mean values with different letters above the quantitative values are significantly different (*p* < 0.05) according to Duncan’s multiple range test (mean ± standard deviation, *n* = 2).

**Table 4:** HPLC-ELSD and GC-FID area % for hexane layers after sequential recrystallisation (4 °C followed by −20 °C), and the final purified product from the transesterification reaction product.

		Area %			
		HPLC		GC	
		Ascorbyl-6-O-Esters	Fatty Acid	Ascorbyl-6-O-Oleate	Ascorbyl-6-O-Palmitate
Hexane layers after recrystallisation	4 °C	NA	NA	90.5 ± 0.3	9.5 ± 0.3
	−20 °C	NA	NA	91.0 ± 0.6	9.0 ± 0.6
Final purified product		94.3 ± 0.1	5.7 ± 0.1	91.1 ± 0.2	8.9 ± 0.2

Mean values with different letters above the quantitative values are significantly different (*p* < 0.05) according to Duncan’s multiple range test (mean ± standard deviation, *n* = 2). NA = not analysed.

## Data Availability

The original contributions presented in this study are included in the article. Further inquiries can be directed to the corresponding author(s).
